# Accuracy and clinical relevance of the single-lead Apple Watch electrocardiogram to identify atrial fibrillation

**DOI:** 10.1016/j.cvdhj.2022.10.004

**Published:** 2022-12-15

**Authors:** Shari Pepplinkhuizen, Wiert F. Hoeksema, Willeke van der Stuijt, Nicole J. van Steijn, Michiel M. Winter, Arthur A.M. Wilde, Lonneke Smeding, Reinoud E. Knops

**Affiliations:** ∗ Amsterdam UMC Location University of Amsterdam, Heart Center, Department of Cardiology, Amsterdam, The Netherlands; † Amsterdam Cardiovascular Sciences, Heart Failure and Arrhythmias, Amsterdam, The Netherlands

**Keywords:** Single-lead ECG, Atrial fibrillation, Accuracy, Wearables, Apple Watch

## Abstract

**Background:**

The Apple Watch (AW) is the first commercially available wearable device with built-in electrocardiogram (ECG) electrodes to perform a single-lead ECG to detect atrial fibrillation (AF).

**Methods:**

Patients with AF who were scheduled for electrical cardioversion (ECV) were included in this study. The AW ECGs were obtained pre-ECV and post-ECV. In case of an unclassified recording, the AW ECG was obtained up to 3 times. The 12-lead ECG was used as the reference standard. Sensitivity, specificity, and kappa coefficient were calculated.

**Results:**

In total, 74 patients were included. Mean age was 67.1 ± 12.3 years and 20.3% were female. In total 65 AF and 64 sinus rhythm measurements were obtained. The first measurement with the AW showed a sensitivity of 93.5% and specificity of 100% (κ = 0.94). A second measurement resulted in a sensitivity of 94.6% and specificity of 100% (κ = 0.95). A third measurement resulted in a sensitivity of 93% and a specificity of 96.5% (κ = 0.90). Adjudication of unclassified recordings by a physician reduced the total unclassified recordings from 27.9% to 1.6%, but also reduced the accuracy. The kappa coefficient for unclassified single-lead ECGs was 0.58.

**Conclusion:**

The single-lead ECG of the AW shows a high accuracy for identifying AF in a clinical setting. Repeating the recording once decreases the total of unclassified recordings; however, a third recording resulted in a lower accuracy and the occurrence of false-positive measurements. Unclassified results of the AW can be reduced by physicians’ interpretation of the single-lead ECG; however, the interrater agreement is only moderate.

## Graphical abstract

**Figure:**
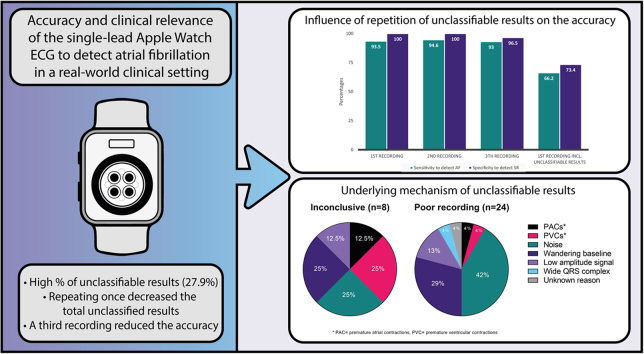

**Key Findings**

• The Apple Watch single-lead electrocardiogram (ECG) showed a high accuracy to identify atrial fibrillation (AF) and sinus rhythm when not including the high percentage of unclassifiable recordings.
• Repeating the recording once decreased the total of unclassified recordings; however, a third measurement resulted in a lower accuracy and the occurrence of false-positive measurements. Our results therefore suggest to repeat only once.
• As physicians’ interpretation of unclassified single-lead ECGs did reduce the total number of unclassifiable results but also lowered the accuracy, the 12-lead ECG remains necessary to confirm diagnoses of AF.

## Introduction

Silent atrial fibrillation (AF) is associated with the appearance of ischemic stroke, which can be significantly reduced with initiation of adequate antithrombotic treatment.^1^^,^^2^ The potential of semicontinuous electrocardiogram (ECG) monitoring outside the clinical setting for the detection of silent AF is promising, but accuracy studies on commercially available devices are limited. The Apple Watch (AW) (Apple Inc, Cupertino, CA) was the first FDA-approved wearable device with built-in ECG electrodes into the watch face to perform a single-lead ECG to detect AF.^3^ The ECG sensors are an addition to the photoplethysmogram sensor, which was also available in previous models. Combining ECG and photoplethysmogram sensors increases the specificity, thereby reducing the risk of false-positives and unwarranted doctor’s visit.^4^

**Figure 1:**
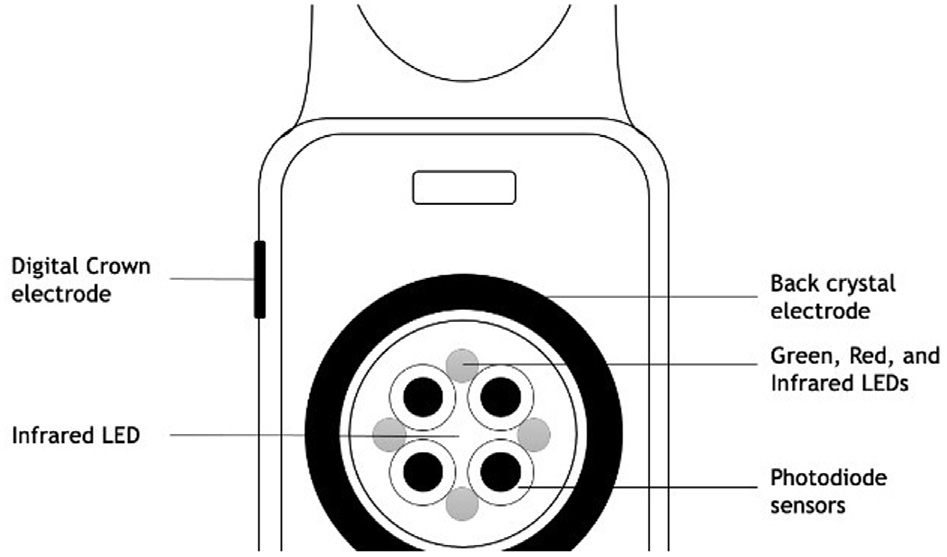
Apple Watch (Apple Inc, Cupertino, CA) sensors.

The ECG electrodes are located in the digital crown and on the back of the AW (Figure 1). To create a single-lead ECG, corresponding with lead I of the 12-lead ECG, active participation of the watch carrier is needed by positioning the index finger of the opposite hand on the crown. Alongside the single-lead ECG, adjudication of the heart rhythm is given by a notification. These notifications include AF, sinus rhythm (SR), low or high heart rate, and inconclusive or poor recording. Inconclusive measurements, according to the manual of Apple Inc, can be caused by the presence of cardiac implantable electronic device (CIED), signs of other arrhythmias or heart conditions, or certain physiological conditions that result in a low-amplitude signal.

Studies on the accuracy for the detection of AF with the AW are inconclusive. Apple Inc’s most recent validation study showed a high specificity and sensitivity (99.3% and 98.5%) and a total unclassifiable rate (inconclusive + poor recordings) of 8%.^5^ To the best of our knowledge, only 2 independent studies studied the accuracy of the AW ECG measured with the newest ECG sensor. The study of Seshadri and colleagues^6^ included post–cardiac surgery patients and compared the AW ECG results with telemetry interpretations. They reported a sensitivity of 41%, specificity of 100%, and inconclusive rate of 31%. The recent study by Abu-Alrub and colleagues^7^ reported a sensitivity of 87%, specificity of 86%, and 9.5% unclassifiable results.

Both studies interpreted the unclassified records as false results when calculating the accuracy, in contrast to Apple Inc’s validation study. The high inconclusive rates and/or potentially higher false-positive rates in the general population may result in a higher demand on the healthcare system and can create unnecessary anxiety in the users.

The discrepancy in results and the increase in use of the AW by the public shows the urgency for more independent clinical accuracy studies.^8^ In this study we aim to describe the accuracy of the AW ECG in a clinical setting and the potential to improve the accuracy by repetition and interpretation of the single-lead ECGs by a physician. In patients scheduled for cardioversion for AF, patients’ measurements before and after cardioversion can be used as their own control group to evaluate the AF detection algorithm.

## Methods

### Study design and population

This was a prospective, nonrandomized, single-center observational study to evaluate the accuracy and interrater agreement of the single-lead ECG of the AW. Patients scheduled for elective electrical cardioversion (ECV) for AF in our center between February and June 2021 were asked for participation. All patients were included in our tertiary hospital. Inclusion criteria were 18 years of age or older, scheduled for elective ECV, and willing and able to provide written informed consent. Patients were excluded when supraventricular tachycardia other than AF were registered on the 12-lead ECG before ECV. This study was approved by the Medical Ethical Committee. The study was conducted in accordance with the Declaration of Helsinki.

### Study materials

The Apple Watch, series 6, with watchOS software 7.2 or 7.3 and ECG algorithm version 2.0 (Apple Inc) was used. The AW application notifications are as follows: AF (up to 150 beats per minute [bpm]), SR, low or high heart rate (<50 bpm or >150 bpm), and inconclusive and poor recording. Inconclusive, low, or high heart rate and poor recordings are combined in this study as unclassifiable recordings.

### Measurements

The AW was positioned on the left wrist and patients were instructed to rest their arm on the table or their legs and keep their arms still during the recording. A test run for correct positioning of the finger was done by each patient before the actual measurements and a member of the research team was present to assure proper measurements. The AW ECG was obtained during AF before ECV and during SR after ECV. If ECV was unsuccessful and SR was not achieved, no AW ECG post-ECV was obtained. When patients arrived in SR, only the pre-ECV SR recording was obtained. All patients who underwent ECV received propofol as anesthetic. The post-ECV SR recording was taken when the patient was fully awake and able to eat, drink, and answer questions clearly. The AW ECG recordings were taken immediately after the first 12-lead ECG recording to prevent interference of both signals. The patients were continuously monitored to avoid a change in rhythm between the AW ECG and 12-lead ECG recordings. In case of an inconclusive or poor recording notification, the AW ECG recording was obtained up to 3 times. The 12-lead ECGs and AW ECGs were blindly adjudicated by 2 physicians (S.P. and W.S.). In case of disagreement, a third physician adjudicated the ECGs. In case of disagreement between all 3, agreement was reached by consensus.

### Endpoints

The main endpoint is the sensitivity to detect AF and the specificity to detect SR of the AW ECG application before and after repetition of unclassified results. Outcome of the 12-lead ECG was used as the reference standard. Secondary endpoints are the kappa coefficient between the AW notifications and 12-lead ECGs; the total rate of inconclusive, low, or high heart rate and poor recordings; the sensitivity and specificity after interpretation of the unclassified ECGs by a physician; and the interrater agreement of the AW ECG between 2 physicians.

### Statistical analysis

Continuous variables were tested for normality with histogram interpretation and Kolmogorov-Smirnov test. Normally distributed data are presented as means with standard deviations. Non–normally distributed data are presented as medians with interquartile ranges. Categorical variables are presented in percentages. The sensitivity and specificity were calculated from contingency tables. The agreement between the single-lead ECG and 12-lead ECG was measured using Cohen’s kappa. Cohen’s kappa between 0.4 and 0.6 was interpreted as moderate interrater agreement, from 0.6 to 0.8 as substantial, and above 0.8 as almost perfect.^9^ Correction for partly paired data was done showing comparison with only 1-sided single measurements pre-ECV and is shown in the Supplemental Table 1. Statistical significance was set at an alpha level of 0.05. Statistical analyses were performed using IBM SPSS Statistics version 26.

## Results

### Patient selection and baseline characteristics

A total of 74 patients were included in this study. Mean age was 67.1 ± 12.3 years, 20.3% were female, 18.9% had a CIED, and the mean body mass index was 28.1 kg/m^2^. Patient characteristics are presented in Table 1. A total of 12 patients did not undergo an ECV, of which 9 (75%) patients had SR on arrival, 2 (16.7%) patients had no adequate anticoagulation, and 1 (8.3%) patient had decompensated heart failure. ECV was performed in 62 patients, with successful conversion to SR in 55 patients. In 6 patients the ECV was unsuccessful and 1 patient had a junctional rhythm after ECV. In total 129 AW ECG measurements were recorded, of which 64 were SR and 65 AF.

**Table 1:** Patients’ characteristics

Characteristics	Result (N = 74 patients)
Age ± SD (years)	67.1 ± 12.3
Female	15 (20.3%)
BMI ± SD (kg/m^2^)	28.1 ± 6.0
CHA_2_DS_2_-VASc score [IQR]	3.0 [1.75–4.0]
Use of anticoagulation	74 (100%)
Vitamin K antagonists	13 (17.6%)
Direct oral anticoagulants	61 (82.4%)
CIED in situ	14 (18.9%)
Pacemaker	5 (6.8%)
ICD	9 (12.2%)

BMI = body mass index; CIED = cardiac implantable electronic device; ICD = implantable cardioverter-defibrillator; IQR = interquartile range.

### Accuracy of the AW single-lead ECG notifications

The AW ECG notification showed a sensitivity of 93.5% to detect AF and a specificity of 100% to detect SR (κ = 0.94) after the first recording (Table 2). The positive predictive value after the first measurement was 100% and the negative predictive value was 94%. A second recording in patients with inconclusive or poor recordings (n = 32) raised sensitivity to 94.6% and specificity remained 100% (κ = 0.95). A third recording in patients who had an inconclusive or poor recording 2 times in a row (n = 15) resulted in a sensitivity of 93.0% and specificity of 96.5% (κ = 0.90). When including the unclassifiable results as false measurements after the first recording the sensitivity was 66.2% and the specificity was 73.4% (κ = 0.53) (Table 3). For 94.4% (34/36) of the unclassified measurements, the physicians were able to adjudicate the heart rhythm based on the AW ECG. When including the physician-adjudicated unclassifiable recordings the sensitivity to diagnose AF was 89.2% and the specificity to detect SR was 93.8% (κ = 0.83) (Table 4).

**Table 2:** Single-lead electrocardiogram notifications compared to physician-interpreted 12-lead electrocardiogram

		12-lead ECG	SR	Total
		AF		
Single-lead ECG notifications	AF	43	0	43
	SR	3	47	50
	Unclassifiable	19 (14.7%)	17 (13.2%)	36 (27.9%)
	HR <50 beats/min	1 (0.8%)	3 (2.3%)	4 (3.1%)
	Inconclusive	4 (3.1%)	4 (3.1%)	8 (6.2%)
	Poor recording	14 (10.9%)	10 (7.8%)	24 (18.6%)
Total		65	64	129

AF = atrial fibrillation; ECG = electrocardiogram; HR = heart rate; SR = sinus rhythm.

**Table 3:** Overview of the influence of repeated measurements, including unclassifiable results, implanted cardiac device, and paced rhythms, on the accuracy of the single-lead electrocardiographic Apple Watch notification

	First recording (N = 129)	Second recording (N = 129)	Third recording (N = 129)	First recording incl. unclassifiable results (N = 129)	First recording excl. patients with CIED (N = 104)	First recording excl. patients with paced rhythm (N = 113)
Sensitivity, AF	93.5%	94.6%	93.0 %	66.2%	97.5%	95.5%
Specificity, SR	100%	100%	96.5 %	73.4%	100%	100%
% Inconclusive	6.2%	3.9%	3.9%	-	5.8%	5.3%
% Poor recording	18.6%	7.8%	4.7%	-	17.3%	17.7%

AF = atrial fibrillation; CIED = cardiac implantable electronic device; Excl. = excluding; HR = heart rate; Incl. = including; SR = sinus rhythm.

**Table 4:** Single-lead electrocardiogram notification plus unclassifiable recordings interpreted by physicians compared to 12-lead electrocardiogram

		12-lead ECG		Total
		AF	SR	
Single-lead ECG notification plus interpretation of unclassifiable recordings by physicians	AF	58	3	61
	SR	6	60	66
	Unclassifiable	1 (0.8%)	1 (0.8%)	2 (1.6%)
	Poor recording	1 (0.8%)	1 (0.8%)	2 (1.6%)
Total		65	64	129

AF = atrial fibrillation; ECG = electrocardiogram; SR = sinus rhythm.

In total, 18.9% (n = 14, 25/129 recordings) of the patients had a CIED, of whom 9 patients (16/129 recordings) had a paced rhythm on the 12-lead ECG. When excluding patients with a paced rhythm on the 12-lead ECG, the sensitivity increased to 95.5% and specificity to 100% (κ = 0.95). Excluding all patients with a CIED (n = 14) further increased the sensitivity, to 97.5% sensitivity to detect AF and 100% specificity to detect SR (κ = 0.97) (Table 3).

### Repetition of inconclusive or poor recordings

After the first recording, 6.2% (8/129) of all recordings were inconclusive and 18.6% (24/129) were poor recordings. After the second recording, 3.9% (5/129) of all recordings were inconclusive and 7.8% (10/129) were poor recordings. After the third measurement, 3.9% (5/129) of all recordings were inconclusive and 4.7% (6/129) were poor recordings (Table 3). Reasons for inconclusive measurements and poor recordings after the first recording, interpreted by the adjudicating physicians, are shown in Table 5.

**Table 5:** Underlying mechanism for inconclusive or poor recordings

	Inconclusive (N = 8)	Poor recording (N = 24)
Premature atrial contraction	1 (12.5%)	1 (4.2%)
Premature ventricular contraction	2 (25%)	1 (4.2%)
Low-amplitude signal	1 (12.5%)	3 (12.5%)
Wide QRS complex	0	1 (4.2%)
Noise	2 (25%)	10 (41.7%)
Wandering baseline	2 (25%)	7 (29.2%)
Unknown	0	1 (4.2%)

Excluding patients with a CIED reduced the total unclassifiable results to 26.9% (28/104), inconclusive measurements to 5.8% (6/104), and the total poor recording rate to 17.3% (18/104). After exclusion of patients with only a paced rhythm, the total unclassifiable results were reduced to 26.5% (30/113), the inconclusive measurements rate was reduced further to 5.3% (6/113), and the poor recording rate increased to 17.7% (20/113) (Table 3). When the physician-adjudicated unclassifiable AW ECG recordings were included, the total unclassifiable recording rate decreased from 24.8% (34/129) to 1.6% (2/129) (Table 4).

### Interrater agreement of the AW ECG

The kappa coefficient of agreement between 2 independent physicians adjudicating all single-lead ECG measurements was 0.69. For the unclassifiable recordings specifically, the kappa coefficient was 0.58.

## Discussion

This is the first study to look at the underlying mechanism of unclassifiable results and the influence of repetition of unclassifiable results on accuracy of the single-lead ECG notification of the AW. In this study, the single-lead ECG of the AW showed a high accuracy to detect AF, with a sensitivity of 93.5% and a specificity of 100% (κ = 0.94). However, the percentage of unclassified recordings was high, 27.9%, and when the unclassified results were included as false results the sensitivity and specificity were significantly lower (66.2% and 73.4%). Repeating the recording once decreased the total of unclassified recordings and increased the sensitivity. A third measurement resulted in a lower accuracy and the occurrence of false-positive measurements.

After inclusion of unclassifiable recordings, the sensitivity in this study was higher compared to the study of Seshadri and colleagues.^6^ This may be owing to the use of telemetry, instead of 12-lead ECG, as the reference standard to detect AF. Also the difference in study population, as Seshadri and colleagues included patients post thoracic surgery, may have had an influence. The lower sensitivity and specificity for the total first measurements in our study compared to the study of Abu-Alrab and colleagues^7^ can be explained by the higher percentage of unclassifiable measurements in our study (27.9% vs 9.5%). This cannot be explained by the fact that we also included patients with a CIED, as after exclusion of patients with a CIED the unclassifiable rate was still 26.9%.

Interpretation of the single-lead ECG by a physician showed that 2 of the major underlying causes of inconclusive and poor recordings were noise and a wandering baseline. Nerves for the ECV procedure might have had an effect on measurement quality; however, as no difference was observed in total unclassifiable results by noise or a wandering baseline before and after ECV, this effect was most likely minimal. Moreover, as all patients in our study got clear instructions, and rested their arm on the table or their legs, it is unclear what caused the noise or wandering baseline. The high percentage of unclassifiable results raises questions on the reliability of the algorithm of the single-lead ECG of the AW, especially for use outside a clinical setting where there are no clear instruction and observations by a physician on the execution of the recording.

Excluding patients with a paced rhythm decreased the inconclusive rate more than excluding all patients with a CIED. This makes it more likely that paced rhythms influence the inconclusive rate instead of the presence of a CIED and may suggest that patients with a CIED without pacing can use the single-lead AW ECG without risk of higher inconclusive results. However, the number of patients with a CIED in this study is too small to draw any conclusion, and more studies with a higher volume of CIED patients are necessary to explore the exact effect of a CIED on the accuracy of the single-lead ECG notifications.

This study showed that the majority of unclassified single-lead AW ECGs can be classified to SR or AF by a physician. However, the interrater agreement between 2 physicians adjudicating the unclassified single-lead ECGs was moderate and reduced the accuracy. This questions the position of the single-lead AW ECG in the clinical pathway, as the 12-lead ECG remains necessary as a reference standard to confirm the heart rhythm.

A limitation of this study is that it was performed in a population with a high prevalence of AF, resulting in a high a priori pretest probability. An estimated high false-positive rate in the general population can potentially lead to an increased demand on the healthcare system. Another limitation is the low number of female patients included. Also, the use of wearables declines with age and is highest in the age group <50 years and therefore, only by age, not the at-risk group for ischemic stroke.^10^ In combination with the high rate of unclassifiable measurements, we need to question if screening for AF should be performed in the general population or in patients with risk factors for ischemic stroke. Patients with risk factors are probably the only ones to benefit from adequate treatment, and careful selection of these patients can prevent overtreatment. Until we have more population-based data on potential benefit, physicians should be conservative in actively promoting the use of the AW in the general population.

The HEARTLINE study, a randomized study in which patients above 65 years of age are randomized to use the AW ECG app, will further investigate the reliability of the AW as a screening tool for AF detection in the general population.^11^

## Conclusion

The AW single-lead ECG showed a high accuracy for the identification of AF and SR. However, the percentage of unclassifiable results was high, especially for poor recordings, which raises questions on the value of the AW for AF screening in the general population. Repeating the recording once decreased the total of unclassified recordings; however, a third measurement resulted in a lower accuracy and the occurrence of false-positive measurements. Our results therefore suggest that inconclusive and/or poor recordings should not be repeated more than once to avoid the risk of false-positives. Including patients with a CIED did not increase the rate of inconclusive recordings, although more studies are needed to confirm this finding. As physicians’ interpretation of unclassified single-lead ECGs did reduce the total number of unclassifiable results but also lowered the accuracy, the 12-lead ECG remains necessary to confirm diagnoses of AF and the position of the single-lead ECG in the clinical pathway is not clear yet.
