# Supplementary material for: Accuracy and clinical relevance of the single-lead Apple Watch electrocardiogram to identify atrial fibrillation
Source: Cardiovasc Digit Health J. 2022 Dec 15;3(6 Suppl):S17–22. doi: 10.1016/j.cvdhj.2022.10.004 (PMC9795256; doi:10.1016/j.cvdhj.2022.10.004)
Supplement: Supplemental Table 1 [file mmc1.docx]

Appendix

Supplemental Table 1. Single-lead ECG notifications compared to physician interpreted 12-lead ECG prior to ECV only

|  | 12-lead ECG interpretation by physician | | | |
| --- | --- | --- | --- | --- |
|  |  | AF | SR | Total |
| Single-lead ECG notifications | AF | 43 | 0 | 43 |
|  | SR | 3 | 7 | 10 |
|  | Unclassifiable | 19 | 2 | 21 (28.4%) |
| Total |  | 65 | 9 | 74 |

Sensitivity 93.5% (43/46), specificity 100% (7/7), kappa coefficient 0.79.
